# The Antioxidative Action of ZTP by Increasing Nrf2/ARE Signal Pathway

**DOI:** 10.1155/2019/5421528

**Published:** 2019-03-10

**Authors:** Liang Yang, Bing Xu, Chunxu Yuan, Zhi Dai, Yong Wang, Qiongya Li, Qifan Yang, Nuomin Li, Hong Qing

**Affiliations:** ^1^School of Life Science, Beijing Institute of Technology, Beijing 100081, China; ^2^College of Life Sciences & Research Center for Resource Peptide Drugs, Shaanxi Engineering & Technological Research Center for Conversation & Utilization of Regional Biological Resources, Yanan University, Yanan 716000, China; ^3^China Resources Sanjiu Medical & Pharmaceutical Co., Ltd. Shenzhen 518110, China

## Abstract

So far, more than 25,000 brain diseases have been shown to be related to oxidative stress. Excessive free radicals and reactive oxygen species (ROS) can attack cells resulting in dysfunctional proteins, lipids, and nucleic acid, finally leading to imbalance of energy metabolism, cell death, gene mutation, and immune reaction. Therefore oxidative stress plays an important role in neuronal diseases. As a traditional Chinese medicine, Zhengtian Pill (ZTP) was reported to have the ability to reduce the blood viscosity of migraine model rats, with increased beta-endorphin, serotonin, adrenaline, and dopamine in brain tissue. Moreover ZTP can effectively accelerate blood circulation and attenuate blood coagulation. However, the molecular mechanisms of ZPT are still unclear. Through the behavioral test we found that ZTP can significantly improve depression-like behavior induced by LPS when rat was treated with ZTP (L 0.17 g/kg, M 0.34 g/kg, and H 0.7 g/kg) intraperitoneal injection once a day for 30 consecutive days. And ZTP can resist oxidative stress (>72 h) for a longer time. And ZTP can promote the levels of ATP and SOD and reduce the levels of ROS and MDA in the brain. At the same time, ZTP can have antioxidant stress through increasing the expression level of Nrf2/HO-1/P38. These results show that ZTP may be a potential antioxidant stress drug for variety of diseases associated with oxidative stress injury.

## 1. Introduction

Oxidative stress has been known for more than 30 years. More than 25,000 brain diseases have currently been shown to be related to oxidative stress. Oxidative stress mainly includes the imbalance between the oxidation and the antioxidant systems in cells and tissues, resulting in excessive free radicals and reactive oxygen species (ROS). ROS are considered to be the main toxic substance produced by aerobic metabolism [1]. Excess ROS products can attack cells to produce dysfunctional proteins, lipids, and nucleic acids, which can lead to a loss of metabolism energy, the alteration of cell signaling and cell cycle, gene mutations, changes in cellular transport, a decrease of the overall biological activity, or the activation of inflammation [2, 3]. Studies have shown that oxidative stress is associated with different kinds of diseases. Researchers have reported that ROS can enhance oxidative stress and inflammation, thereby damaging insulin sensitivity in diabetes [4, 5]. At the same time oxidative stress plays an important role in the development of diabetic complications, including microvascular and diabetic cardiovascular related diseases [6]. In the pathological study of stroke, it is found that oxidative damage associated with ischemia is a major cause of tissue necrosis [7]. Increased levels of oxidative stress aggravate metabolic syndrome in obese patients [8]. Recently, researchers have found that ROS is the key factor for chronic neurodegenerative diseases that cause age-related vision loss [9]. Abnormal expression of oxidative stress can lead to schizophrenia [10]. Oxidative stress and inflammation associated with many diseases can promote each other in pathological processes [11]. Also, oxidative stress can lead to neurodegenerative disorders such as Parkinson and Alzheimer's disease [6, 12–14].

Zhengtian Pill (ZTP), a proprietary Chinese medicine, passed the State Food and Drug Administration Ordinance in 1987 [15]. Zhengtian Pill can reduce the blood viscosity of migraine rat model, increase the expression levels of beta-endorphin, serotonin, adrenaline, and dopamine in brain tissue and can effectively promote blood circulation and reduce coagulation [16]. Zhengtian Pill mainly contains three kinds of volatile oil substances include protocatechuic acid, ferulic acid, and ligustilide. However, the pharmacodynamic mechanisms of Zhengtian Pill are still unclear. The main reason is that Zhengtian Pills is a drug containing 15 kinds of medicinal herbs [17]. It also requires a large amount of research to determine which components play a role in specific diseases. This experiment aims to reveal whether the ZTP has the ability to resist oxidative stress.

In recent years, many studies have found that lipopolysaccharide (LPS) can induce oxidative stress and inflammatory reactions and other pathological features. It has been reported that a single injection of LPS can induce a sustained neuroimmune response leading to the loss of neurons in the substantia nigra. LPS can also cause motor dysfunction associated with neurodegenerative diseases, systemic oxidative stress, and dopaminergic neuron degeneration [18–20]. Previous studies found that LPS can induce anxiety, learning and memory disorders, and other depression-like behaviors [21–23]. In this work, we used LPS to establish an oxidative stress model and studied the antioxidant ability of Zhengtian pills. Behavioral results showed that Zhengtian pills could effectively antagonize depression-like behaviors caused by LPS. At the same time, Zhengtian pills can decrease the level of malondialdehyde and hydrogen dioxide and augment the level of Superoxide Dismutase and ATP in a dose-dependent manner. Meanwhile, it was found that Zhengtian Pill could synergistically resist the oxidative stress level by activating Nrf2 and HO-1. These studies would help us to better understand the mechanism of the effect of Zhengtian pills.

## 2. Materials and Methods

### 2.1. Animals

The KM mice (20-23 g) were provided by the experimental animal center, National Institutes for Food and Drug Control (Beijing, China, No SCXK (Jing) 2014-0013). All mice were kept under standard conditions at 22°C and a 12-h light/dark cycle with free access to food and water. Sixty KM mice were randomly divided into six groups: normal control group, model group, Vitamin E group (100 mg/kg), ZTP low dose group (L 0.17 g/kg), ZTP moderate dose group (M 0.34 g/kg), and ZTP high dose group (H 0.7 g/kg). Mice were administered drugs or saline solution (normal and model groups) orally once a day for 8 days. After the last gavage, mice in the model group and medicine treatment groups were infected with LPS 5mg/kg (Sigma L2880 USA) [19]. The behavioral test started the next day after the administration was completed. Mice were anesthetized with 60 mg/kg Pentobarbital Sodium (i.p) and sacrificed after behavioral testing. All experiments were carried out under the guidance of the Beijing animal ethics Association and the ethics committee of Beijing Institute of Technology.

### 2.2. Drugs

Zhengtian pills (ZTP) are Chinese patented medicine comprised of 15 medicinal herbs (Z44020711): rhizoma Chuanxiong, Radix Angelicae Sinensis, Flos Carthami, Herba Asari, Radix Saposhnikoviae, Radix Angelicae Pubescentis and Radix Aconiti Lateralis Preparata, etc. [24]. Vitamin E (T3251), LPS (L2880), and Pentobarbital Sodium were purchased from Sigma Chemical Co., (USA).

### 2.3. Behavioral Test

#### 2.3.1. Sucrose Water Preference Experiment

All mice were trained for sugar water adaptability for 4 days. First, after 24 h of putting two bottles of sugar water on the cage, one bottle of sugar water was replaced with daily drinking water for another 24 h and then they were fasted for 24 h. Each cage was filled with an equal amount of sugar water and daily drinking water. The mice were free to drink for 1 h and finally measured the consumption and we calculated the consumption ratio for evaluation (sugar water/ (sugar water + daily drinking water) ×100).

#### 2.3.2. Forced Swimming Test (FST)

The forced swimming test was similar to the protocol described elsewhere [25]. Briefly, the mice were placed in a glass cylinders (height:40 cm; diameter:10 cm;containing 30 cm of water at 25°C) for 6 minutes. A mouse was judged to be immobile when it ceased struggling and remained floating motionless in the water. The immobility time was recorded during the last 4 min of the 6 min testing period.

#### 2.3.3. Novelty-Suppressed Feeding Test (NSFT)

The novelty-suppressed feeding test is a sensitive assessment for evaluating anti-anxiety-like behavioral deficits [26]. After fasting for 24 h, each one was placed in a plastic box (100 cm×50 cm×25 cm) with a food in fixed position. The latency was recorded within 5 min when the mice began eating. Moreover, the home-cage food consumption was recorded in 10min to evaluate the effects of drugs on the feeding drive.

#### 2.3.4. Tail Suspension Test (TST)

The tail suspension test was based on the method of Steru [27]. The mice were suspended 40 cm above the floor through an adhesive tape and placed approximately 1cm from the tip of the tail. The immobility time was recorded during the last 4 min of the 6 min testing period.

#### 2.3.5. Determination of Oxidative Stress Biomarkers in Brain Tissue

The levels of hydrogen peroxide (H_2_O_2_), malondialdehyde (MDA), and superoxide dismutase (SOD) in brain tissue were measured by different kits (A064-1, A003-1, A001-3) provided by NanjingJiancheng. Adenosine triphosphate was tested by Elisa kit (CEA349Ge, Cloud-clone crop, USA).

#### 2.3.6. Estimation of Brain Mitochondrial Complex Enzyme Activity Level

Mitochondrial complex enzyme I, II, III, and IV activity levels were detected by mitocheck complex activity assay kits (Nos. 900930, 700940, 700950, and 700990 Cayman USA).

#### 2.3.7. Western Blot

The brain tissues were lysed using RIPA buffer (Cat#R0010, Solarbio, China) and sonicated to obtain proteins solution. Proteins concentration was determined by the Bradford method. 10% SDS-PAGE gel was used to separate proteins, which was then transferred to the PVDF membrane (CAT. NO. ISEQ00010). The membranes were blocked with 5% skimmed milk in TBS tween-20 (TBST), then incubated with the primary antibodies [Nrf2 (ab62352, abcam), P38MAPK (ab32142, abcam) heme oxygenase-1 (HO-1) (ab68477, abcam)], diluted in TBST at 4°C overnight, and then incubated with the HRP-conjugated secondary antibody at room temperature for 2 h. The images were detected using Bio-Rad ChemiDoc XTS+ system [28].

#### 2.3.8. Immunofluorescence Staining

Brains were fixed in 4% paraformaldehyde for 48 hours and then transferred to 20% sucrose for 24 h and later were placed in 30% sucrose. The sectioned 20 *μ*m was used for immunofluorescence staining. For (HO-1) immunofluorescence staining, the sections were blocked in a phosphate buffer containing 5% horse serum for 2 h, and then the sections s were transferred into an intermixture containing rabbit anti-HO-1 antibody ((1/100) HBSS +0.03% TritonX-100+1.5%FBS) for 12 h at normal temperature. The sections were incubated in secondary antibody for 2 h at RT (1/500, ab150074, abcam, USA). For P38 staining, sections were permeabilized with phosphate buffer containing 0.5%TritonX-100 for 1 hour at room temperature. The sections were incubated with buffer containing 5% horse serum for 2 h and then transferred into rabbit anti-P38 mixture ((1/250) PBS+0.3% TritonX-100+3% horse serum) for 12 h at 4°C. The usage of secondary antibody is the same as that of HO-1.

### 2.4. Statistical Analysis

Data are expressed as mean ± standard error of mean (SEM). The one-way analysis of variance (ANOVA) was used for data analysis. Difference associated with* P*< 0.05 was considered statistically significant.

## 3. Results

### 3.1. Effects of ZTP on Depressive-Like Behavior and Body Weight Gain in Mice

**Figure 1:**
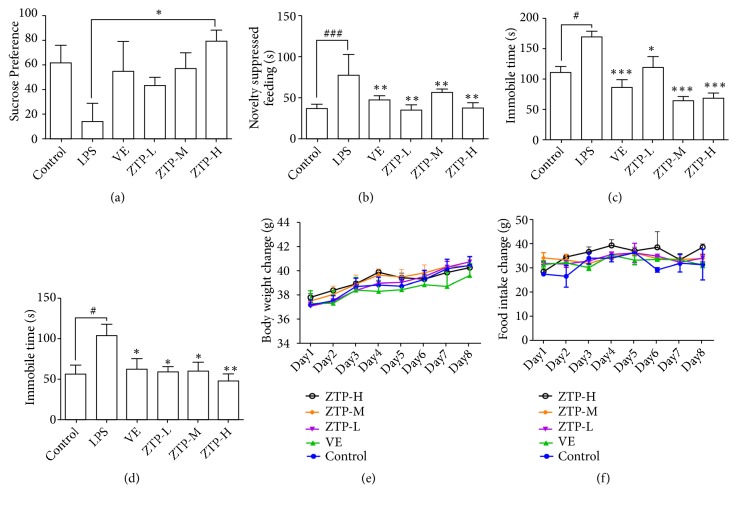
*Animal behavior test and body weight feeding record in the mice*. (a) Sugar Preference Experiment: the mice were divided into 6 groups' control, LPS, VE, and ZTP (H 0.7 g/kg, M 0.34 g/kg, and L 0.17 g/kg). (b) Experimental inhibition of food intake in 6 groups of mice. (c) Forced swimming experiment in 6 groups of mice. (d) Suspension test of 6 groups of mice. (e) Eight consecutive days to detect changes in body weight in mice. (f) Eight consecutive days of food intake in mice. ^#*∗*^*p* < 0.05, ^##*∗∗*^*p* < 0.01, ^###*∗∗∗*^*p* < 0.01, ^#^Control* VS* LPS, ^*^LPS* VS* Treatment (VE/ZTP-L/ZTP-M/ZTP-H).

In order to investigate whether the ZTP has an effect on body weight gain, we tested the weight of mice for eight days in a row. The result showed that different treatments had no significant effect on the body weight gain of mice (Figure 1(e)). At the same time, we also recorded the daily food intake of each cage mouse. The results showed that the diet of each group was not affected by the administration (Figure 1(f)). These results indicated that the different doses of the drugs had no effect on the body weight gain of the mice. Previous studies have shown that LPS-induced oxidative stress can cause depression-like behavior in mice [29]. In the sucrose preference test (Figure 1(a)), no difference was observed between baseline preference levels for sucrose water before LPS stress. LPS stress remarkably decreased sucrose preference. ZTP high-dose treatment group can significantly block the development of sucrose anhedonia caused by LPS compared with the LPS group. In novelty-suppressed feeding test (NSFT), the LPS (5 mg/kg) significantly exhibited a longer latency to feed than the control group (*P*<0.01) (Figure 1(b)). The positive drug (Vitamin E) and the different dosage of ZPT (L 0.17 g/kg, M 0.34 g/kg, and H 0.7 g/kg) were significantly decreased compared with the LPS model group [30]. We found that ZTP has significant impact on immobility time in forced swimming test (FST). As shown in Figure 1(c), LPS (5 mg/kg) significantly increased (*P*<0.05) immobility time in the FST compared to the control group, indicating the depressive-like behavior in mice. And the positive drug and different dosage of ZTP (L 0.17 g/kg, M 0.34 g/kg, and H 0.7 g/kg) significantly reduce the LPS-induced immobility time in the FST (L* p*<0.01, M* p*<0.001, and H* p*<0.001). At last, we did a tail suspension test (TST) associated with the desire to survive. As shown in Figure 1(d), LPS (5 mg/kg) significantly* increased* (*p*<0.05) immobility time in the TST compared to the control group. And the different dosage of ZTP (L 0.17 g/kg, M 0.34 g/kg, and H 0.7 g/kg) can* decrease* the immobility time (L* p*<0.05, M* p*<0.05, and H* p*<0.01) in a dose-dependent manner. The above results show that the ZTP treatment could ameliorate the depression-like behavior induced by LPS.

### 3.2. Effects of ZTP on Reactive Oxygen Species (ROS) in Mice Brain

**Figure 2:**
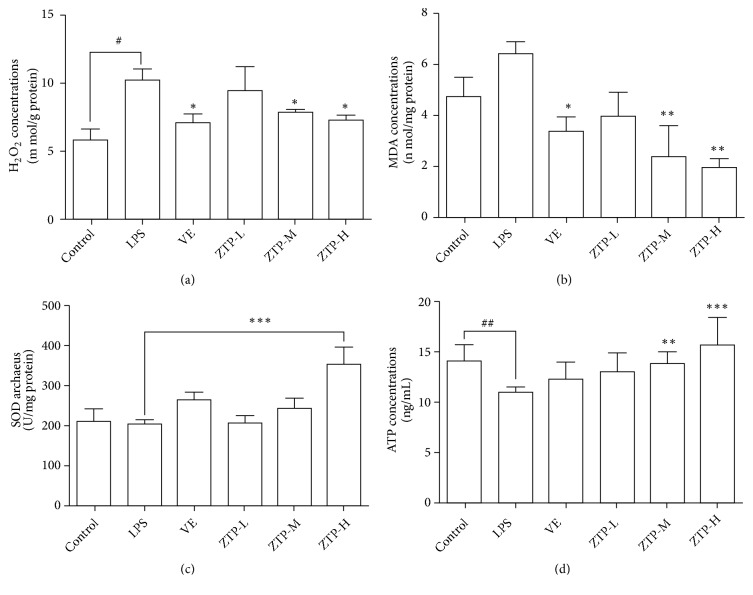
*Detection of relative expression levels of ROS-related factors in mice brain*. (a) The content of hydrogen peroxide in brain tissue of 6 groups of mice. (b) Lipid peroxide (LPS) content in brain tissue of 6 groups of mice. (c) The content of superoxide dismutase (SOD) in brain tissue of six groups of mice. (D) The content of adenosine triphosphate (ATP) in brain tissue of six groups of mice. ^#*∗*^*p* < 0.05, ^##*∗∗*^*p* < 0.01, ^###*∗∗∗*^*p* < 0.01, ^#^Control* VS* LPS, ^*^LPS* VS* Treatment (VE/ZTP-L/ZTP-M/ZTP-H).

Studies demonstrated that oxidative stress will cause serious biological effects when the concentration of H2O2 in tissue reaches a certain level (>100 nM) [31]. Based on the above reasons, we measured the levels of H_2_O_2_ in brain tissue of mice, and the results showed that the concentration of H_2_O_2_ in brain tissue of LPS group was significantly higher than that of control group (*P*<0.05) (Figure 2(a)). The VE and different dosage of ZTP (M, H* P*<0.05) significantly decreased the concentration of H_2_O_2_ in brain. Malondialdehyde (MDA) is one of the commonly used biomarkers for studying oxidative stress levels [32]. The concentration of MDA can reflect the degree of lipid peroxidation and indirectly detect the extent of cell damage. To investigate whether the ZTP can have antioxidative stress, we measured lipid peroxidation (MDA). As shown in Figure 2(b), the ZTP-M and ZTP-H groups remarkably upregulated the concentration of MDA compared with LPS mice (M, H* P*<0.01). Previous studies have shown that high Superoxide dismutase (SOD) activity in the body can improve the body's ability to resist oxidative stress [33]. The result indicated that the SOD activity in the mitochondria of mice from the ZTP-H group was significantly higher than LPS group (*P*<0.001) (Figure 2(c)). The results displayed a significant increase ATP concentration in brain of ZTP-M and ZTP-H groups compared to the LPS (M* P*<0.01, H* P*<0.001) (Figure 2(d)). Summarizing the experimental results, we found that ZTP can increase energy metabolism and peroxidase activity to protect the organism from excessive free oxygen damage. At the same time, this drug can reduce the level of free radicals and its induced lipid peroxide, thus regulating the balance of ROS.

### 3.3. ZTP Promotes Expression Level of HO-1 and P38 Proteins in Mice Brain

**Figure 3:**
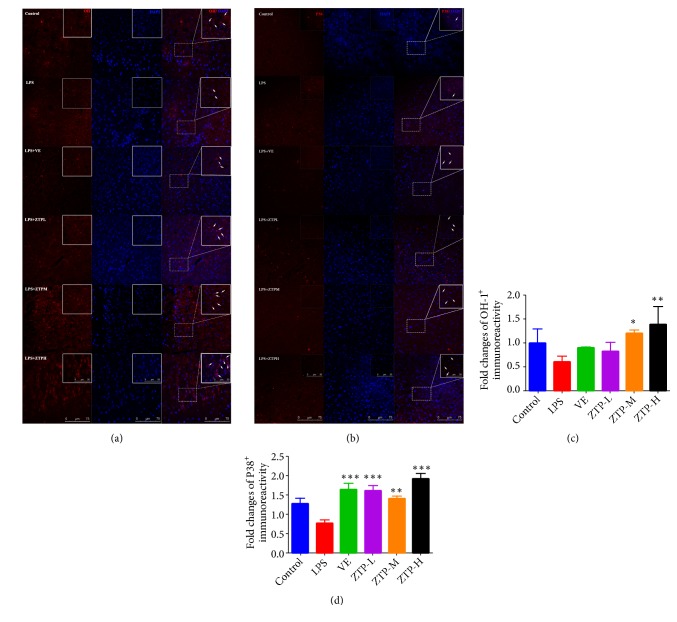
*Expression level of HO-1 and P38 proteins in cortex*. (a, b) Positive immunofluorescence staining of HO-1 and P38 cells in the cortex in control, LPS VE, ZTP-L, ZTP-M, and ZTP-H groups. (c, d) Fold changes of positive HO-1 and P38 immunoreactivity. ^#*∗*^*p* < 0.05, ^##*∗∗*^*p* < 0.01, ^###*∗∗∗*^*p* < 0.01, ^#^Control* VS* LPS, ^*^LPS* VS* Treatment (VE/ZTP-L/ZTP-M/ZTP-H).

In order to investigate the mechanism of ZTP against oxidative stress, we used immunofluorescence to detect the expression levels of HO-1 and P38 in the cortex of mice. It has been reported that HO-1 was activated under oxidative stress and its expression level is upregulated. [34]. The results indicated that compared with the LPS group, the expression level of HO-1 was significantly increased in ZTP group (M* P*<0.05, H* P*<0.01) (Figures 3(a) and 3(c)). At the same time, the expression level of nonphosphorylated P38 protein in mouse brain also increased significantly under ZTP treatment (M* P*<0.01, L H* P*<0.001) (Figures 3(b) and 3(d)). Immunohistochemistry results revealed an enhanced resistance to oxidative stress in the ZTP groups that was likely due to the upregulation of the proteins levels of HO-1 and p38.

### 3.4. ZTP Treatment Affects the Nrf2/HO-1/P38 Signaling Pathway in the Brains

**Figure 4:**
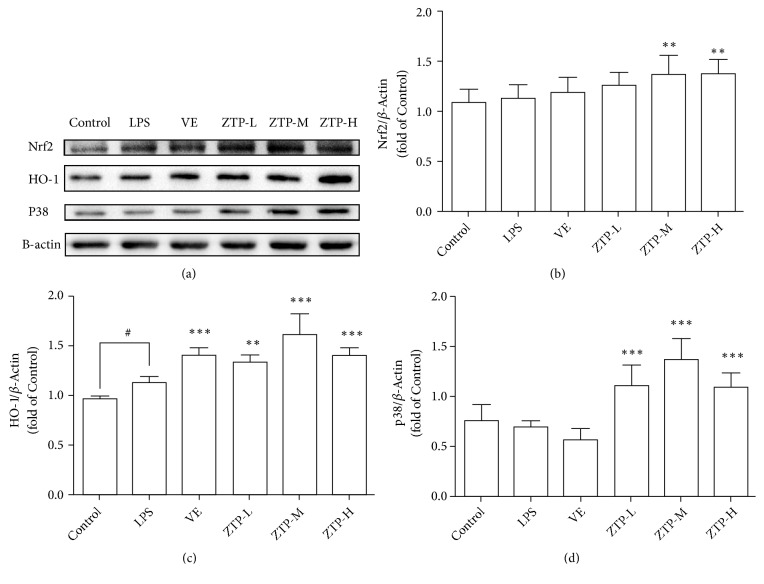
*Expression level of Nrf2/HO-1/P38 proteins in mice*. (a) Representative Western blot showing Nrf2, HO-1, P38, and *β*-Actin in hippocampal, respectively, in 6 groups mice. (b) Relative immunoreactivity of Nrf2 normalized to *β*-Actin. (c) Relative immunoreactivity of HO-1 normalized to *β*-Actin. (d) Relative immunoreactivity of P38 normalized to *β*-Actin. ^#*∗*^*p* < 0.05, ^##*∗∗*^*p* < 0.01, ^###*∗∗∗*^*p* < 0.01, ^#^Control* VS* LPS, ^*^LPS* VS* Treatment (VE/ZTP-L/ZTP-M/ZTP-H).

Numerous studies have reported that the organism reduces oxidative stress by upregulating Nrf2/HO-1 expression levels [35–37]. Moreover it has also shown that P38 can promote the activation of Nrf2 [38]. So we tested the expression level of Nrf2/HO-1 and P38 in the brains of different groups (Figure 4(a)). As shown in Figure 4, the protein level of Nrf2 was remarkably increased in ZTP-M and ZTP-H group compared with that in the LPS group (M, H* P*<0.001) (Figure 4(b)). The expression of HO-1 protein was also obviously increased under ZTP treatment (L* P*<0.01, M H* P*<0.001) (Figure 4(c)). Researches show that, with the increase of oxidative stress, the expression level of p-P38 is also on the rise and the increase of p-P38 causes the death of cells [39, 40]. As shown in Figure 4(d), we found that the expression level of P-38 of ZTP treatment groups was notably increased compared to LPS group (L M H* P*<0.001) (Figure 4(d)). In general, the mechanism of ZPT antioxidant stress may be achieved by increasing the expression level of Nrf2/HO-1/P38.

## 4. Discussion

Several studies reported that reducing the ATP content in the brain of AD animal models could aggravate oxidative stress effects [41].

In this study, we reported that ZTP ameliorates the depressive-like behaviors caused by oxidative stress, reduces the expression of ROS-related factors in brain tissue, and regulates the expression levels of oxidative stress-related proteins, which include Nrf2, HO-1, and P38.

In this experiment, we wanted to understand whether ZTP can actively prevent oxidative stress injury and still have a sustained effect after stopping administration. First, we gave the mice a gradient dose of ZTP (L 0.17g/kg, M 0.34 g/kg, and H 0.7 g/kg) for a period of time (8 days) and then used LPS to induce oxidative stress. Thereafter, ZTP was not administered anymore, from behavioral testing to molecular detection. The experimental results show that ZTP has the ability to continue to resist oxidative stress (>72 h) in a long time window, and it is clear that ZTP has a good linear trend in the dose structure-activity relationship, which proves that ZTP-H has a significant antioxidant effect.

Different experimental results reflect the multipathway antioxidant effects of ZTP, such as increasing ATP and SOD content in brain tissue, reducing the level of ROS related markers H_2_O_2_ and MDA brain tissue. This suggests that ZTP regulates the related enzymes and energy metabolism on the one hand and, on the other hand, it can scavenge oxygen peroxides and harmful metabolic lipid peroxides.

The changes of ATP concentration can directly reflect the level of oxidative stress. It is reported that more ATP is needed to maintain the energy balance when LPS induces ROS to stimulate the host immune response [42]. The increase of reactive oxygen will lead to a rapid decrease of ATP in cells, indicating that oxidative stress will result in abnormal expression in key proteins regulatory in glucose metabolism [41]. SOD also plays an important role in the defense of ROS damage which is a key factor to eliminate ROS. Studies have shown that SOD can protect the organism against ROS induced by viruses, bacteria, parasites, and physical chemistry [42]. In addition, with the increase of SOD concentration, the levels of ROS and lipid peroxides decrease and apoptosis decreases [33, 41, 42].

In our research, we also tested mitochondrial enzymatic activity. The result implies that ZTP can improve mitochondrial function stability under oxidative stress. As shown in Figure S1, the ZTP groups showed higher enzymatic activity than the LPS group, especially in enzyme I and enzyme III (ZTP-H* vs* LPS enzyme I>48%, III>64%) (Figure S1).

Many studies have reported that synergistically upregulating expression levels of Nrf2 and HO-1 can protect gene damage from cell damage and apoptosis [36]. Others have also found that increased ROS levels leading to increased levels of p-P38, inducing DNA damage, and further production of more reactive oxygen species [38]. Our result indicated that ZTP can reduce oxidative stress levels and increase the expression level of nonphosphorylated P38, which suggests that ZTP may inhibit ROS activation by increasing the expression of P38. Overall, this research found that ZTP can regulate gene expression levels, increase the expression levels of related proteins against oxidative stress, and improve oxidative stress injury. These results show that ZTP may be a potential antioxidant stress drug for variety of diseases associated with oxidative stress injury.

## 5. Conclusions

In conclusion, this experiment found that Zhengtian Pill has antioxidative stress, relieves depression-like behavior, and improves oxidative stress through Nrf2 signaling pathway.

## Data Availability

All data generated or analysed during this study are included in this published article (and its supplementary information files).

## Abbreviations

ROS: Reactive oxygen species
ZTP: Zhengtian pill
LPS: Lipopolysaccharide
FST: Forced swimming test
NSFT: Novelty-suppressed feeding test
TST: Tail suspension test
H_2_O_2_: Hydrogen peroxide
MDA: Malondialdehyde
SOD: Superoxide dismutase
HO-1: Heme oxygenase 1
Nrf2: Nuclear fac-tor erythroid 2-related factor 2
p38: p38 MAPK
TBST: Tris buffered saline and tween-20
ATP: Adenosine triphosphate.

## Supplementary Materials

Supplementary Materials Detection of brain mitochondrial complex enzyme activity level. The authors investigated mitochondrial enzyme activity in the mice cortex. Mitochondrial complex enzyme I, II, III, and IV activity levels were detected by mitocheck complex activity assay kits (Nos. 900930, 700940, 700950, and 700990 Cayman USA). The authors tested mitochondrial enzymatic activity. The result implies that ZTP can improve mitochondrial function stability under oxidative stress. As shown in Figure S1, the ZTP groups showed higher enzymatic activity than the LPS group, especially in enzyme I and enzyme III (ZTP-H vs MED enzyme I>48%, III>64%) (Figure S1).
