# Supplementary material for: The Antioxidative Action of ZTP by Increasing Nrf2/ARE Signal Pathway
Source: Evid Based Complement Alternat Med. 2019 Mar 10;2019:5421528. doi: 10.1155/2019/5421528 (PMC6431467; doi:10.1155/2019/5421528)
Supplement: Supplementary Materials — Detection of brain mitochondrial complex enzyme activity level. The authors investigated mitochondrial enzyme activity in the mice cortex. Mitochondrial complex enzyme I, II, III, and IV activity levels were detected by mitocheck complex activity assay kits (Nos. 900930, 700940, 700950, and 700990 Cayman USA). The authors tested mitochondrial enzymatic activity. The result implies that ZTP can improve mitochondrial function stability under oxidative stress. As shown in Figure S1, the ZTP groups showed higher enzymatic activity than the LPS group, especially in enzyme I and enzyme III (ZTP-H vs MED enzyme I>48%, III>64%) (Figure S1). [file 5421528.f1.docx]

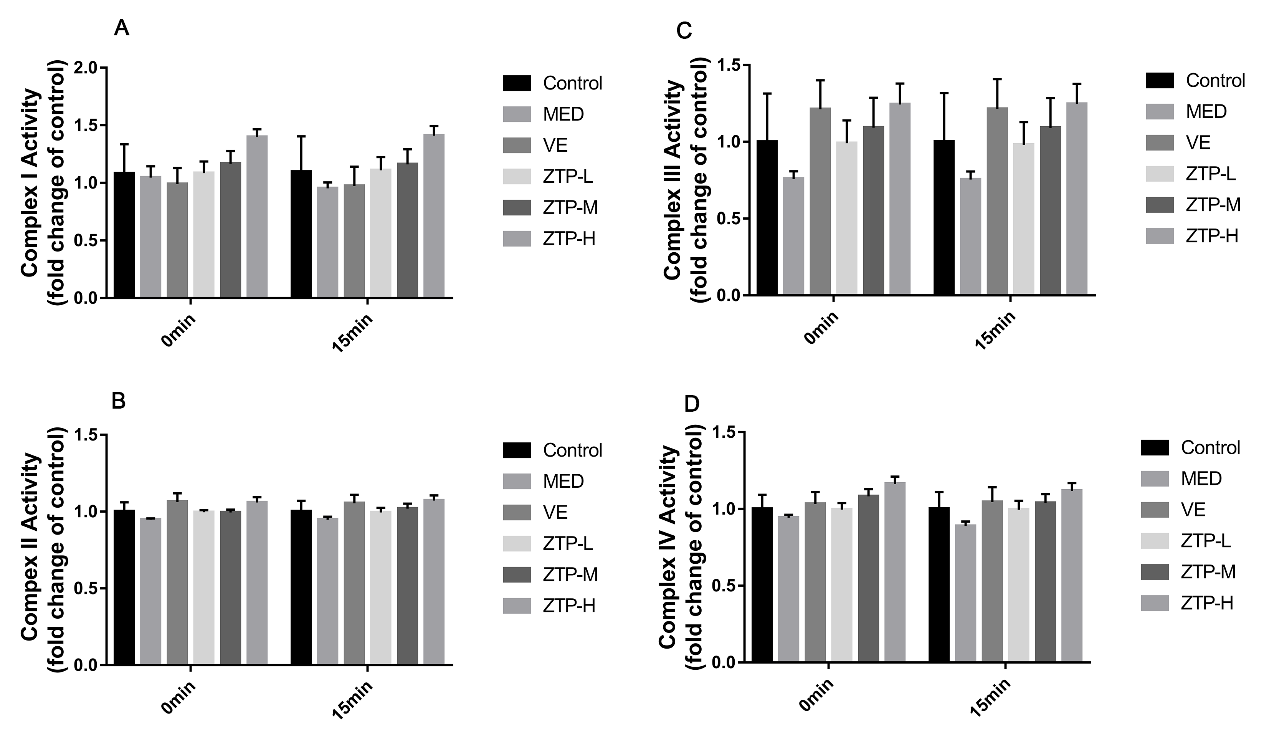


**Figure S1 Test of mitochondrial enzyme activity in brain tissue**

1. Viability testing of mitochondrial complex enzyme I in brain tissue of 6 groups at two time points (0 min, 15 min). (B) The activity of mitochondrial complex enzyme II in 6 groups of mice brain tissues was measured at two time points. (C) Viability testing of mitochondrial complex III in brain tissue of 6 groups at two time points. (D) Viability testing of mitochondrial complex IV in brain tissue of 6 groups at two time points. Compared with MED group, by two-way ANOVA; mean + S.E.M. in bar graphs.

**Description of supplementary material file**

***Detection of brain mitochondrial complex enzyme activity level***

We investigated mitochondrial enzyme activity in the mice cortex. Mitochondrial complex enzyme I, II, III, IV activity levels were detected by mitocheck complex activity assay kits (No 900930, 700940, 700950, 700990 Cayman USA).

we tested mitochondrial enzymatic activity. The result imply that ZTP can improve mitochondrial function stability under oxidative stress. As shown in Fig.S1, the ZTP groups showed higher enzymatic activity than the LPS group, especially in enzyme I and enzyme III (ZTP-H *vs* MED enzyme I>48%, III>64%) (Fig. S1).
